# Non-utilization of Public Health Care Facilities by Women of Reproductive Age Group: A Cross-Sectional Study in an Urban Area of Central India

**DOI:** 10.7759/cureus.17212

**Published:** 2021-08-16

**Authors:** Sudarshan C Wagh, Ujwala U Ukey, Sarita K Sharma

**Affiliations:** 1 Department of Community Medicine, Government Medical College & Hospital, Nagpur, IND

**Keywords:** health care, non utilization, women, reproductive age group, urban, cross-sectional study

## Abstract

Introduction

Non-utilization of public health care facilities by women in India is one of the crucial concerns when ensuring universal health coverage. However, contrary to the fact that women need more health care assistance throughout their lifespan, there is a considerable lack of awareness among them, and this is a major contributor to their unwillingness to use these services.

Methods

A community-based descriptive cross-sectional study was conducted among women of the reproductive age group (15-49 years) in an urban field practice area of a tertiary health care center in central India. Data were collected for two months by interview technique using a semi-structured questionnaire. Data were analyzed using Epi Info version 7.2.2.6 (Centers for Disease Control and Prevention, Atlanta, Georgia) software.

Results

Of the total 132 women, 77 (58.33%) respondents were aware of the availability of public health care facilities in their area of residence. Despite this, only 59 (44.69%) were utilizing the services. Non-utilization of public health care facilities was significantly more in those belonging to upper socioeconomic status (chi-square = 14.36, *p* < 0.05 at a degree of freedom [df] = 2). The common reasons being lack of personal attention, cleanliness, and overcrowding at these facilities.

Conclusion

Even though a substantial population in central India cannot afford private or corporate health care services, the utilization of public health care facilities has not been up to the mark. Overall, most of the subjects were aware of the facilities available to them. This awareness, however, did not match with the utilization of such facilities. Less than half of the women were utilizing the public health care facilities.

## Introduction

India has a mixed health care system, inclusive of public and private health care service providers [1]. Although a large number of hospitals exist in cities, the public health infrastructure remains grossly inadequate in urban areas. National Health Policy (NHP) 2002 has expressed the requirement for focusing on the urban population. The National Urban Health Mission (NUHM) has perhaps been at a reduced pace to take off and gain momentum for fulfilling the desired objectives [2,3]. It has often been stated that public health care is a basic service. However, on the contrary, the system in place is ailing and unresponsive [4,5]. This has led to its non-utilization, specifically in urban areas.

In the majority of states in India, the health systems work towards removing the barriers to health care utilization with a target to attain equitable access to health care facilities. To realize this goal, it is imperative to make a systematic assessment of prevailing patterns of utilization of health care services, extent, and determinants of non-utilization at an individual and societal level [6,7]. In the current situation, women in India face a multitude of health issues that, if not addressed promptly and within time, can ultimately affect our economy [8]. The World Health Organization (WHO) states that today’s women’s health has become an urgent priority, yet the data surrounding this issue are limited and often unreliable [9,10]. Urban India has a higher concentration of health care providers, yet not everyone has easy access to health care [11].

Globally, the bulk of the health care is provided by women, both in the formal health care setting and the informal sector, as well as the home. Yet women’s own needs (particularly of women in the reproductive age group) for health care are poorly addressed, especially in developing countries like India [12]. Closer monitoring of women’s opinions about the reasons for non-utilization is an important implication not only for understanding their mentality, but also to devise ways to overcome them in the long run, and eventually uplift the health status of women in India. With this background, the current study was planned with the objectives to determine the non-utilization of public health care facilities by women of the reproductive age group and also to identify the possible reasons for the non-utilization.

## Materials and methods

Study design and setting

A community-based descriptive cross-sectional study was conducted in an urban area under the tertiary health care facility in Nagpur, a city in central India for two months i.e., from February 1, 2020, to March 31, 2020.

Study population

Women in the reproductive age group in the field practice area of the urban health training center of a tertiary care facility were the study population. Only one person was interviewed from each home that was visited. If there were more than one woman of reproductive age group in the same household, the elder one was interviewed. In the present study, all the women in the reproductive age group, i.e., 15 to 49 years, were included. The women not willing to participate in the study were excluded.

Sample size and sampling

For calculation of sample size, the formula applied was Z^2^pq/d^2^, where Z is a constant value, p denotes prevalence, q denotes 1-p, and d is the allowable error obtained from the precision. Based on the study of Dalal et al. [13], considering the non-utilization of public health care facilities to be 58%, the estimated minimum sample size came out to be 170 (with a 95% confidence interval and a 15% degree of relative precision). The researchers went to the entry point of the urban health training center where a pen was rotated to decide the direction for moving in the study area and then tossed a coin to choose the right or left side of the street. Further, the last two digits of a randomly selected currency note were considered to decide the number of footsteps and thus, the first household was selected randomly. Women from every alternate household were interviewed (based on the inclusion and exclusion criteria) in this community-based survey. The same procedure was applied on each day of data collection, taking care that the survey is not carried in the same area again. Thus, a representative sample from the study group was collected by systematic random sampling.

Study tool

For this survey, a pre-designed, semi-structured questionnaire with open and close-ended questions on various aspects of utilization of public health care facilities was used as the study tool. A pilot survey was conducted on 15 subjects by using the pro forma. Based on the results of the pilot survey, necessary changes were made in the pro forma. The final survey pro forma consisted of four sections as follows: section 1 - sociodemographic background; section 2 - awareness of public health care facilities; section 3 - perceptions of public health care facilities; and section 4 - reasons for non-utilization of public health care facilities.

Ethical aspects

Ethical clearance was obtained from the Institutional Ethics Committee (IEC) vide letter number 1982EC/Pharmac/GMC/NGP regarding the conduct of the study. Participation in the study was entirely voluntary. The participants were apprised of the nature and purpose of the study and assured of complete anonymity and full confidentiality.

Data collection and statistical analysis

Data were collected by interview technique using a pre-structured questionnaire, with a consent form appended to it. These interviews were collected in the vernacular language (i.e., Hindi or Marathi) of the subject. Data were entered in Microsoft Excel software and were analyzed using Epi Info version 7.2.2.6 (Centers for Disease Control and Prevention, Atlanta, Georgia) software. Percentage, mean, and standard deviation (SD) were calculated from the data. Suitable statistical tests were applied to obtain the level of significance.

## Results

Sociodemographic characteristics of respondents

During the present survey, 170 households were approached to achieve the estimated sample, of which 132 consented to participate (response rate of 77.64%). All subjects were females belonging to the reproductive age group in the selected urban area. The mean age of the study participants was 24.59 years (SD = 9), with a minimum age reported as 17 years and a maximum of 47 years. Most of the study participants were from the age group of 20-24 years (n = 79, where n indicates the number of those particular responses; 59.84%) followed by the age group of 15-19 years (n = 26; 19.69%). Those in the age group of 25-29 years were eight (6.06%); 30-34 years were four (3.03%); 35-39 years were six (4.54%); 40-44 years were five (3.78%), and 45-49 years were six (4.54%). Out of the 132 responses received, 117 (88.63%) women were unmarried followed by 13 (9.84%) married; 1 (0.75%) widowed and 1 (0.75%) divorced. The questionnaire also determined the percentage of nuclear (n = 96; 72.72%), joint (n = 20; 15.15%), and three-generation (n = 16; 12.12%) families. The size of the family showed an inclination towards three to six members (n = 111; 84.09%) as compared to more than six (n = 16; 12.12%), and less than three (n = 5; 3.78%) members. Table 1 summarizes the sociodemographic particulars of the study participants.

**Table 1:** Sociodemographic characteristics of the study participants (n = 132)

Categorical variables		Frequency, n (%)
Educational qualification	Professional or honors	15 (11.36)
	Graduate	54 (40.90)
	Intermediate or diploma	20 (15.15)
	High school certificate	42 (31.81)
	Illiterate	1 (0.75)
Employment status	Employed	59 (44.69)
	Homemakers	73 (55.30)
Socioeconomic status (as per modified Kuppuswamy’s scale)	Upper (I)	25 (18.93)
	Upper middle (II)	66 (50.00)	
	Lower middle (III)	24 (18.18)	
	Upper lower (IV)	16 (12.12)	
	Lower (V)	1 (0.75)	
House	Own	114 (86.36)
	Rented	18 (13.63)
Residential area	Individual house	80 (60.60)
	Flat	33 (25.00)
	Bungalow	14 (10.60)
	Slum	5 (3.78)
Size of family	Less than 3	5 (3.78)
	3-6	111 (84.09)
	Above 6	16 (12.12)
Vehicles owned	Two-wheeler	54 (40.90)
	Car	64 (48.48)
	Both	2 (1.50)
	Others	5 (3.78)
	None	7 (5.30)

An attempt was made to establish the role of socioeconomic status (SES) in the utilization of the public health care facilities by the study participants as shown in Table 2 ahead. The influence of SES of the study participants broadly considered as upper, middle (upper-middle and lower-middle together), and lower (upper-lower & lower together) on the utilization of the facilities was assessed by applying the chi-square test with the degree of freedom (df) as 2. It was observed that there is a significant difference in the utilization of public health care facilities among individuals of different socioeconomic statuses.

**Table 2:** Association between the utilization of public health care facilities and socioeconomic status (n = 132) Chi square = 14.36, *p *= 0.00076 at df = 2. Abbreviations: SES, socioeconomic status; df, degree of freedom.

SES	Utilization of public health care facilities		
	Yes, n(%)	No, n (%)	Total, n (%)
Upper	3 (2.28)	22 (16.66)	25 (18.94)
Middle	49 (37.12)	41 (31.06)	90 (68.18)
Lower	7 (5.30)	10 (7.58)	17 (12.88)
Total	59 (44.70)	73 (55.30)	132 (100)

Awareness of public health care facilities

The responses regarding awareness of women about various aspects of public health care facilities are shown in Table 3. A higher response was recorded for more availability of private hospitals or clinics in nearby 5 km distance as compared to government or corporate hospitals within the same radius. In the present study, 77 (58.33%) respondents expressed that they are aware of the existing public health care facilities in their area of residence. However, 12 (9.09%) study participants were not sure and the remaining 43 (32.57%) study participants did not know that such facilities exist in their area.

**Table 3:** Awareness of public health care facilities (n = 132)

Questions	Yes, n (%)	No, n (%)	Do not know, n (%)
Are there any government or corporation hospitals in the nearby 5 km distance?	109 (82.57)	19 (14.39)	04 (3.00)
Are there any private hospitals or clinics in the nearby 5 km distance?	128 (96.96)	04 (3.00)	00 (0.00)
Are there any government specialty hospitals located in your area?	47 (35.60)	75 (56.81)	10 (7.50)
Are there any general charges fixed by those government hospitals located in your area?	64 (48.48)	15 (11.36)	53 (40.15)
Are you aware of any preventive disease programs conducted by the government hospitals in your area?	69 (52.27)	32 (24.24)	31 (23.48)
Are you aware of the medical camps conducted by the government hospitals in your area?	50 (37.87)	53 (40.15)	29 (21.96)

Perceptions regarding public health care facilities

The perceptions of the study participants regarding public health care facilities were assessed on a five-point Likert scale. These perceptions are represented in Table 4. Most of the women believed that these centers were often (n = 49; 37.12%) or always (n = 26; 19.69%) very over-crowded. They also asserted that the centers were unclean and unhealthy (n = 54; 40.90%), ill-equipped with uncooperative personnel (n = 67; 50.75%) leading to a supply of poor quality of care (n = 58; 43.93%), which was sometimes not fast and prompt (n = 53; 40.15%). Even though the majority of the study participants were aware of the public health care facilities in their area of residence, only 64 (46.04%) were utilizing the services and the remaining 75 (56.96%) were not.

**Table 4:** Perceptions regarding public health care facilities (n = 132)

Perceptions	Never, n (%)	Rarely, n (%)	Sometimes, n (%)	Often, n (%)	Always, n (%)
The public health care facility centers are very over-crowded	4 (3.00)	15 (11.36)	38 (28.78)	49 (37.12)	26 (19.69)
The public health care facility is very unclean and unhealthy	11 (8.33)	14 (10.60)	54 (40.90)	36 (27.27)	17 (12.87)
The public health care facility personnel are very uncooperative	11 (8.33)	30 (22.72)	67 (50.75)	18 (13.63)	6 (4.54)
The public health care facility provided poor quality of care	15 (11.36)	37 (28.03)	58 (43.93)	15 (11.36)	7 (5.30)
The public health care facility provided is not fast and prompt	17 (12.87)	15 (11.36)	53 (40.15)	15 (11.36)	32 (24.24)

Reasons for non-utilization of public health care facilities

**Figure 1:**
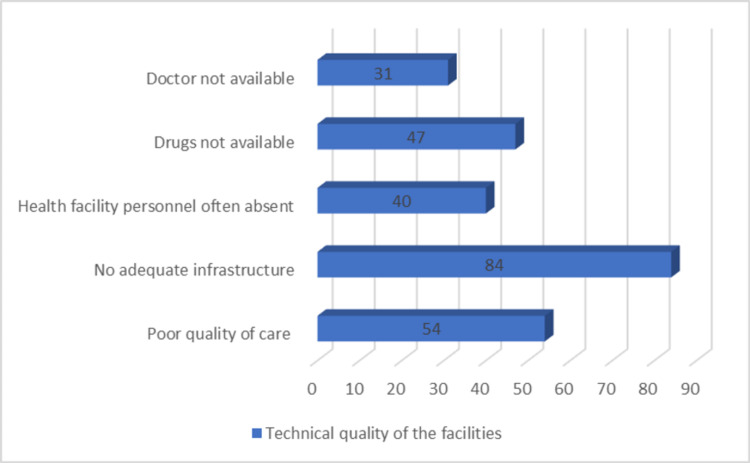
Technical quality of the facilities

**Figure 2:**
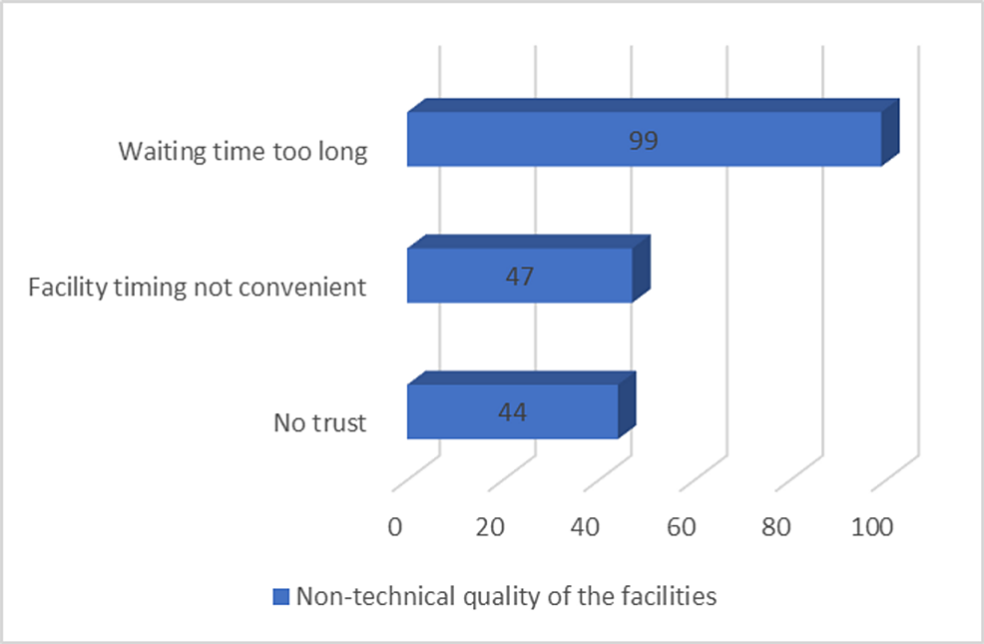
Non-technical quality of the facilities

When inquired about the reasons for non-utilization in terms of access, technical and non-technical qualities of these facilities, about 109 (82.57%) of the total participants enunciated that they had no access to these facilities with the strikingly higher agreement towards the inadequate infrastructures (n = 84; 63.63%) and cumbersome waiting times (n = 99; 75.00%) making them the two most important technical and non-technical reasons for non-utilization, respectively. Several other reasons classified under technical and non-technical reasons recorded as multiple responses have been compiled in Figures 1, 2.

A higher percentage of women also reported that the lack of personal attention (n = 91; 68.93%), modern technology (n = 76; 57.57%), well-furnished and equipped rooms (n = 75; 56.81%) along with poor sanitary conditions (n = 83; 62.87%) were among the topmost problems they faced with public health care facilities, leading to the realization that essentially the health care delivery is lacking followed by medical and general care delivery.

## Discussion

The non-utilization rates of public health care facilities have been on a rise in India for the last few decades [14]. There is a great degree of hue and cry in this context in the Indian health care delivery system. The purpose of the present study was to determine the non-utilization of public health care facilities by women of reproductive age group in an urban area of central India and also to identify the barriers for comprehensive utilization by searching the reasons for the same. The majority of the women were aware of the services that are available in their area of residence. In the present study, the non-utilization of the public health care facilities was observed in more than half of the study participants. This was found to be significantly more in women from the upper SES. These findings are coherent with that of Dalal et al. who observed that non-utilization was present in 58% of their study participants [13].

The present survey revealed varied reasons and barriers among the women of the reproductive age group for non-utilization, specifically lime-lighting the instrumental role of sociodemographic variables, awareness, and societal perceptions regarding the same. Dalal et al. reported a similar relationship among these factors determining the non-utilization [13]. This observation is of vital importance in a meager resource country like India, where the government is always at the blame of the public for inequitable, inaccessible, and poor quality services. This statement is very well-supported by the present study finding of multidimensional approaches to non-utilization. The present study revealed a preference for private health care services over the public by the study participants. This finding is similar to studies from other parts of India [15,16,17]. Among the technical and non-technical aspects, technical aspects have been more specifically found to be lacking at several levels. A similar observation was made in a study, where utilization of public health care services was assessed in households with non-communicable diseases [17]. The present study identified infrastructure deficit, inconvenient, and incompatible facility timings, unnecessarily long waiting times as the stumbling blocks when it comes to a smooth supply of health care. Coincidentally, Dalal et al. and Kujawski et al. also identified them as the root cause [13,17].

Elias et al. brought attention to the lack of human resources viz. absence of doctors, staff, and other health personnel in these facilities when teamed up with the poor quality of medicines as the source of distrust among the masses [18]. These findings are consistent with the present study as it concurs with the reasons for non-utilization viz. dearth of personal attention, scarcity of modern technology, paucity of well furnished, and equipped rooms clubbed with poor sanitary conditions indicating the insufficiency of the system in place in meeting the population’s need [17]. Hence, under these circumstances, there is an urgent need to address the issue of non-utilization which is done through the present study.

Strengths and limitations of the study

An important strength of the present study is that it is community-based and carried out in women in an Indian setup wherein similar studies were not conducted earlier. Overcrowding in these facility centers was one of the major concerns of the study subjects. However, not much attention has been paid to this aspect of health care delivery in other studies. The present study also has certain limitations as it employs a cross-sectional design. Additionally, the study was conducted in an urban area of central India with fewer markers of non-utilization. Hence, results of the present study may not be generalized to the entire country. Further, studies among women of reproductive age group from multiple urban areas with a larger sample need to be carried to substantiate the findings, elucidate utilization patterns, and thereby assist policymakers to formulate recommendations for improvisation especially in the low-income developing countries.

## Conclusions

Even though a substantial population in the modern Indian setup cannot afford private or corporate health care services, the utilization of public health care facilities has not been up to the mark due to various reasons as highlighted in the present study. Overall, most of the study participants were aware of the facilities available to them, preventive disease programs, and medical camps being held in their locality. However, this awareness did not match with the utilization of such facilities. The non-utilization of public health care facilities was significantly more in those with upper socioeconomic status. This issue needs to be addressed promptly with appropriate reforms in health care delivery to uplift the overall health status of women in India.
